# Different Levels of Context-Specificity of Teacher Self-Efficacy and Their Relations With Teaching Quality

**DOI:** 10.3389/fpsyg.2022.857526

**Published:** 2022-06-30

**Authors:** Désirée Thommen, Urs Grob, Fani Lauermann, Robert M. Klassen, Anna-Katharina Praetorius

**Affiliations:** ^1^Faculty of Arts and Social Sciences, Institute of Education, University of Zurich, Zurich, Switzerland; ^2^Institute for Research on Education and School Development, TU Dortmund University, Dortmund, Germany; ^3^Psychology in Education Research Centre, Department of Education, University of York, York, United Kingdom

**Keywords:** teacher motivation, teacher self-efficacy, teaching quality, context-specificity, class-specificity, multilevel regression analyses

## Abstract

On the basis of Bandura’s social cognitive theory, researchers often assume that a teachers’ self-efficacy (TSE) will have a positive effect on teaching quality. However, the available empirical evidence is mixed. Building on previous research into TSE, we examined whether assessing class-/task-specific TSE gives a more accurate indication of the associations between TSE assessments and student-rated teaching quality. The analyses were based on the English sample of the Teaching and Learning International Survey (TALIS) Video Study. Mathematics teachers (*N* = 86) rated their self-efficacy beliefs using generalized task-specific TSE items and class-/task-specific TSE items. Their students (*N* = 1,930) rated the quality of teaching in their math class. Multilevel regression analyses revealed stronger associations between student-rated teaching quality and class-/task-specific TSE than generalized task-specific TSE. We discuss possible reasons for these results and outline the potential benefits of using class-specific assessments for future TSE research.

## Introduction

In research on teacher motivation, self-efficacy is considered a key motivational characteristic of teachers, emphasizing the belief in their own ability to influence student engagement and learning, even when they encounter difficulties (Tschannen-Moran and Woolfolk Hoy, 2001; Klassen and Chiu, 2010). Teacher self-efficacy (TSE) has attracted attention in educational research in recent decades as an important contributor to outcomes such as teacher well-being, and student achievement and motivation (Caprara et al., 2006; Klassen et al., 2011). It is also assumed that teachers with high self-efficacy beliefs perceive themselves as more competent and confident in managing difficult situations in the classroom and this in turn leads to higher-quality teaching (Bandura, 1997; Tschannen-Moran et al., 1998). Empirical findings on the relations between TSE and teaching quality, however, have not been consistent, with studies finding both negative and positive relations between the two constructs (refer to the review by Lauermann and ten Hagen, 2021).

One reason for these inconsistent results could be the varied ways in which the studies conceptualize and assess TSE; the studies differ with respect to their degree of context-specificity (Lauermann and ten Hagen, 2021). Even though self-efficacy beliefs were originally conceptualized as context-specific characteristics, meaning that they could fluctuate depending on the task or situation (Bandura, 1986, 1997), the vast majority of studies have treated TSE as a trait-like characteristic that can be generalized across different teaching contexts (Zee et al., 2016). The different students and classes teachers teach throughout the day are pivotal contextual factors that can contribute to different TSE ratings (Dellinger et al., 2008). To date, only two empirical studies have investigated class-specific TSE evaluations. These studies show that TSE varies considerably across different classes and that this intra-teacher variance is correlated with class-specific characteristics (e.g., class size, achievement levels, and student engagement; Raudenbush et al., 1992; Ross et al., 1996). Therefore, investigating TSE on a general level fails to account for the context-specificity, particularly the class-specificity, of TSE.

It is likely that class-specificity would be particularly relevant for teachers who teach multiple classes (e.g., secondary-level teachers). Recognizing the intra-teacher variance of self-efficacy beliefs across different classes, several researchers have highlighted the need to assess TSE with reference to a specific class (Raudenbush et al., 1992; Lauermann and Berger, 2021; Lazarides et al., 2021). Using class-specific TSE scales should result in comparatively stronger associations with student-rated indicators of teaching quality because individuals’ self-efficacy beliefs are most accurate in predicting corresponding behaviors when measured with a similar level of context specificity as their presumed outcomes (Pajares, 1996; Bandura, 2006; Chesnut and Burley, 2015). Given that teaching quality, which is also a context-specific characteristic, is usually assessed with reference to a specific class (Göllner et al., 2018), class-specific evaluations of TSE could result in more consistent relations with teaching quality than those of generalized TSE scales. A misalignment between generalized TSE and class-specific teaching quality measures could be the reason for the inconsistent results.

A few studies have used class-specific adaptations of established TSE scales (Holzberger et al., 2013; Perera and John, 2020), and these show significant positive associations with teaching quality. However, no study to date has directly compared the predictive effect of TSE scales that use different levels of context-specificity on student-rated teaching quality. A key objective of this study is to conduct comparative analyses of class-/task-specific versus generalized task-specific TSE scales and their associations with teaching quality in the same sample.

In the subsequent sections, we present the conceptualization and presumed classroom implications of TSE from the perspective of social cognitive theory. We then outline the context-specificity of existing TSE measures and demonstrate why it is important to assess TSE with reference to a specific class. Finally, we present the aims of this study.

## Theoretical Background

### Teacher Self-Efficacy and How It Relates to Teaching Quality

Self-efficacy is a key motivational characteristic of teachers and describes the teacher’s judgment of their perceived ability to influence student engagement and learning, even in difficult situations (Tschannen-Moran and Woolfolk Hoy, 2001; Klassen and Chiu, 2010). Research on TSE builds on social cognitive theory (Bandura, 1997), which posits that an individual’s behavior is influenced by the interplay of personal, behavioral, and environmental factors. Specifically, TSE beliefs are shaped by efficacy-building experiences such as mastery experiences (e.g., successful student achievement), vicarious experiences (e.g., observation of a successful behavior of a colleague), verbal persuasion (e.g., positive feedback from a colleague), and physiological activity (e.g., heart rate) (Fackler and Malmberg, 2016). Self-efficacy beliefs not only influence performance, but also goal setting, effort, and perseverance in attaining goals, which then represent new sources of information for an adapted estimation of one’s self-efficacy (Bandura, 1997). This interplay illustrates that self-efficacy beliefs do not refer to actual competences but rather to the self-evaluated levels of competence.

Teacher self-efficacy has garnered increased attention in research on teacher motivation in the past 30 years and appears to be an important factor in teacher development, teaching practice, and student outcomes (refer to the reviews by Klassen et al., 2011; Lauermann and ten Hagen, 2021). There is an assumption that teachers with high levels of TSE are less likely to experience burnout and more likely to be satisfied with their job (e.g., Skaalvik and Skaalvik, 2007). Studies have also found positive relations between TSE and student achievement and motivation (e.g., Caprara et al., 2006). Research further suggests a positive association between TSE and teachers’ classroom behavior. Teachers with a high level of self-efficacy tend to be harder working, more persistent in the face of obstacles, and capable of implementing more challenging and innovative teaching methods (Tschannen-Moran et al., 1998; Klassen and Tze, 2014).

In research on teaching, teaching quality is considered a key determinant of student learning achievement (Hattie, 2009). Over the last decades, various frameworks have been developed to describe pivotal characteristics of teaching quality (for an overview, refer to Praetorius and Charalambous, 2018). Among others, the Three Basic Dimensions of Teaching Quality (TBD) referring to three pivotal characteristics has emerged as being especially useful for describing teaching quality: 1. *Classroom management* – maximizing students’ time on task by coping effectively with disruptions and implementing clear rules and routines. Through effective classroom management, students are provided with disruption-free learning opportunities that can be used for engaged learning processes and activities. Well-organized classroom management environments therefore foster student learning (Seidel and Shavelson, 2007). 2. *Cognitive activation* – encompasses discursive teaching and intensive higher-order thinking, by, for example, providing complex tasks and encouraging problem solving. Cognitive activating teaching aims for a deeper understanding of the learning content and a depth of processing and therefore promotes students’ learning and achievement (Lipowsky et al., 2009). 3. *Student support* – fostering positive and supportive relations between themselves and students, for example, by providing constructive feedback and adopting a positive attitude toward student errors. A supportive classroom climate fosters positive engagement and a feeling of social relatedness, competence, and autonomy, which enhances student motivation (Rakoczy, 2008). For a detailed overview of the three basic dimensions of teaching quality and their assumed effects, refer to the studies by Klieme et al. (2006, 2009). Teaching quality dimensions have often been assessed using student ratings, as they are based on students’ day-to-day classroom experience. These ratings represent a valid, reliable, and cost-effective assessment perspective (Clausen, 2002; Praetorius et al., 2018; Göllner et al., 2021).

Contrary to theoretical expectations, empirical findings on the relations between TSE and the three basic dimensions of teaching quality (classroom management, cognitive activation, and student support) are rather inconsistent across the existing studies (refer to the reviews by Zee and Koomen, 2016 and by Lauermann and ten Hagen, 2021). Hence, studies, which find positive cross-sectional links between TSE and student-rated dimensions of teaching quality, seem to be as common as studies that show no significant relation. For example, in studies by Burić and Kim (2020); Fauth et al. (2019), and Ryan et al. (2015), significant positive cross-sectional links have been found between TSE and the three basic teaching quality dimensions. However, others have not been able to find significant cross-sectional links between TSE and student-rated teaching quality dimensions (e.g., Guo et al., 2012; Jamil et al., 2012). Also, the few longitudinal studies found inconsistent relations between TSE and student-rated teaching quality. While the study by Holzberger et al. (2013) found significant positive relations between TSE and teaching quality dimensions, the two other existing longitudinal studies by Lazarides et al. (2021) and Praetorius et al. (2017) found no significant longitudinal relations. The positive longitudinal effect of cognitive activation and classroom management on student-rated TSE in the study of Holzberger et al. (2013) indicates that TSE may not only be a predictor but also an outcome of high-quality teaching. Considering the importance of teaching quality in research on educational effectiveness, it is important to establish a better understanding of the empirical links between TSE and teaching quality. One reason for the inconsistent findings across the studies could be the various conceptualizations and measurements of TSE used by researchers, which differ with respect to their levels of context-specificity (Lauermann and ten Hagen, 2021; Lazarides et al., 2021).

### Context-Specificity of Teacher Self-Efficacy Measures

The question of what constitute appropriate conceptualizations and measurements of TSE has been a topic of debate for decades (Klassen et al., 2011). Over the years, various conceptualizations and measures have been developed, from general to more specific levels of TSE. Early empirical research mostly treated TSE as a relatively stable, almost trait-like characteristic of teachers that indicated a teacher’s belief in their capabilities (Gibson and Dembo, 1984; Schwarzer et al., 1999). Researchers following this theoretical stance thus treated within-teacher variance in TSE as error-variance (Zee et al., 2016). Generalized measures are not tailored to the teaching process itself but relate to various rather broad areas of teachers’ work (e.g., social interactions with parents). Even though they have commonly been used for studying TSE across different school grades and subjects from 1998 to 2009 (refer to Klassen et al., 2011), these unidimensional measures have been criticized for their lack of predictive validity (Bandura, 1997). This is because the items are often formulated in such a way that is not clear what precisely is being measured. For example, items such as “I can enforce changes within the model project over skeptical colleagues” are ambiguous and fail to specify contextual details. Such a general, undifferentiated, perspective seems particularly problematic, as it does not reflect the many facets of the complex nature of teaching that teachers face in their daily life (Tschannen-Moran et al., 1998). General measures neglect the basic tenets of the social cognitive theory on which TSE is based, which suggests that self-efficacy does not reflect a uniform stable-trait characteristic of a person. Instead, TSE is context-specific, as “some situations require greater skill and more arduous performances, or carry greater risk of negative consequences, than others” (Bandura, 1986, p. 411). Bandura (2006) was therefore critical of “all-purpose” self-efficacy measures, as they do not refer to particular tasks and situations (p. 307).

In early research, the context-specificity of TSE was largely ignored; general TSE ratings with little or no connection to the relevant teaching task or situation were favored (Lazarides and Warner, 2020). Recognizing the drawbacks of general measurements, later researchers started putting a stronger emphasis on the context-specific nature of TSE and developing new measurements (Zee et al., 2016). This resulted in a shift from general to task-specific conceptualizations of TSE. One of the most prominent scales is the Teachers’ Sense of Efficacy Scale (TSES) developed by Tschannen-Moran and Woolfolk Hoy (2001). This scale comprises three fundamental teaching-related tasks in a teachers’ daily life: TSE for classroom management, instructional strategies, and student engagement. The assumption is that a teacher may feel efficacious about, for example, dealing with classroom disruptions, while perceiving himself/herself as less effective in building supportive relationships with the students. The TSES is applicable across different grades and school subjects (Tschannen-Moran and Woolfolk Hoy, 2007; Klassen et al., 2009).

Even though the development of task-specific TSE measurements moved the field toward a more valid approach for assessing the self-efficacy beliefs of teachers by tailoring their items toward specific teaching-related tasks, the vast majority of studies on TSE still implicitly assume that TSE is generalizable across different teaching situations (Dellinger et al., 2008). Researchers following Bandura’s notion that TSE is task and situation-specific argue that TSE fluctuates not only across teaching-related tasks but also across different teaching situations (e.g., Tschannen-Moran and Woolfolk Hoy, 2001; Zee et al., 2016). Dellinger et al. (2008), for example, adhered to the idea that TSE represents a “teacher’s individual beliefs in their capabilities to perform specific teaching tasks at a specified level of quality in a specified situation” (p. 752). Thus, the authors argued that a teacher might experience different levels of self-efficacy across various teaching-related tasks and teaching situations (specific schools, classrooms, and students). A pivotal situational context that varies in teachers’ daily work is the different classes that they teach, as teachers deal with different kinds of environments and challenges in each class (Raudenbush et al., 1992).

There are several reasons why assessing TSE not only via task-specific but also class-specific items, such as the tailoring of TSE items to specific classes, could be productive. First, individual studies have shown that between 21 and 44% of the total variation of teachers’ self-efficacy beliefs reflect within-teacher variation across classrooms (e.g., Raudenbush et al., 1992; Ross et al., 1996). Despite the limitation of TSE being assessed with a single item in both studies, the findings confirm that teachers’ self-efficacy beliefs are not stable and generalizable across different teaching situations but vary across different classrooms. Second, considering the within-teacher variation of TSE across classes is particularly important for research in secondary schools or high schools, where teachers usually have multiple classes. Previous studies that examined TSE at this level used generalized measures for assessing TSE (e.g., Künsting et al., 2016; Praetorius et al., 2017; Burić and Kim, 2020) and therefore failed to consider the class-specificity of TSE. Thus, the evaluation of TSE might be ambiguous and open for interpretation, as it is unclear which class is being referred to Zee et al. (2016). A teacher might answer the same item differently depending on whether they are thinking of a comparatively easy or difficult class. With reference to the four main sources of TSE (refer to section “Teacher Self-Efficacy and How It Relates to Teaching Quality”), external norm criteria such as past or present experiences with a particular class, contextual cues (e.g., classroom characteristics), or references (e.g., class comparisons) might influence teachers when they are reporting their level of self-efficacy toward a specific class (Zee et al., 2018). For example, a teacher might interpret high student achievement in their class as a kind of mastery experience, indicating their teaching success, which then might positively affect the nature of their self-efficacy beliefs (Fackler and Malmberg, 2016). By contrast, the same teacher might assess TSE differently if the items are related to a different class with which they experience frequent stress and frustration in class. It is therefore important that TSE items refer to a specific class. Third, assessing TSE with class-specific instead of generalized measures also seems beneficial in terms of its predictive validity, as self-efficacy scales are deemed most predictive when measured as context-specific as possible (Bandura, 1997, 2006). A recent meta-analysis confirmed that generalized TSE measures suffer from low predictive validity and fail to uncover relations with context-specific outcomes (Chesnut and Burley, 2015). This study concurs with a recent review by Lauermann and ten Hagen (2021) and indicates that context-specific TSE measures have a higher magnitude of relations with contextualized outcomes than generalized measures. A misalignment of context level between predictor and outcome might therefore have contributed to the inconsistency of the findings of studies investigating the relation between TSE and teaching quality to date. While TSE is usually assessed in general terms, items for teaching quality dimensions are mostly context-specific and tailored to a specific class because teaching quality is considered to be a classroom-level phenomenon (Göllner et al., 2018; Aditomo and Köhler, 2020). Therefore, assessing TSE on a class-specific level might increase predictive validity and strengthen associations with class-specific teaching quality and several researchers have recently called for a more context-specific assessment of TSE (e.g., Bandura, 2006; Zee et al., 2018; Lazarides and Warner, 2020).

To the best of our knowledge, only two studies have included class-specific adaptations of established and ad hoc TSE scales (refer to the review by Lauermann and ten Hagen, 2021) to study the relations between teaching quality dimensions and TSE (Holzberger et al., 2013; Perera and John, 2020). The introductory sentence of both of those self-efficacy questionnaires referred to a target class, aligning them to class-specific teaching quality. The increased validity of such class-specific TSE measurements could have contributed to the significant relations found in both studies between class-specific TSE and teaching quality dimensions. In contrast, a study by Praetorius et al. (2017) that used the same TSE measures as Holzberger et al. (2013), but without tailoring the instrument to a specific class, found that the relations were not significant. These preliminary findings support the assumption that context-specific judgments of TSE have higher predictive power for relations with contextualized outcomes (Chesnut and Burley, 2015). Despite the growing literature and the call for more context-specific TSE measures, no study has yet conducted a direct comparison of how different levels of context-specificity in TSE relate to teaching quality. A direct comparison would enable, for the first time, an analysis of whether class-specific TSE measures have advantages for assessing teaching quality. This might go some way toward clarifying the findings of inconsistent relations between TSE and teaching quality.

### The Present Study

Encouraged by the previous findings of context-specific TSE (Bandura, 2006; Chesnut and Burley, 2015), this study aimed to investigate TSE not only in relation to a specific teaching-related task, but also to a specific class. We extend the study by Holzberger et al. (2013), which referred to a specific class but neglected the task-specificity of TSE, as they used the general measure of Schwarzer et al. (1999). We have not only incorporated the generalized task-specific TSE measure of Tschannen-Moran and Woolfolk Hoy (2001), but also tailored the introductory sentence and all items to a specific class. This should align the TSE measurement more closely to teaching quality. By directly comparing two TSE scales with different levels of context-specificity (the generalized task-specific TSE scale vs. the adapted class-/task-specific TSE scale), we also aim to explore their predictive validity. Specifically, the study explores the following research questions:

1. How is class-/task-specific TSE related to the three basic dimensions of teaching quality?
2. How do the relations to teaching quality dimensions differ between class-/task-specific TSE and generalized task-specific TSE?

Based on previous results, we expect that class-/task-specific TSE will be positively related to classroom management [H1a], cognitive activation [H1b], and student support [H1c]. We also expect that the relations of class-/task-specific TSE and classroom management [H2a], cognitive activation [H2b], and student support [H2c] are significantly stronger than the ones with generalized task-specific TSE.

## Materials and Methods

### Participants and Procedure

Data were drawn from the Teaching and Learning International Survey (TALIS) Video Study. The main data collection of the study was conducted in 2017 and 2018 (OECD, 2020a). This study is based on country-level data from England. The English data sample was selected because it included the planned sample size of *N* = 85 classes, and the instrument quality of the target scales was judged to be sufficient (this was not the case for some of the other countries). In the final sample of this study, ratings of *N* = 86 mathematics secondary teachers from 78 schools (all state-funded, 74% located in urban areas) and their *N* = 1,930 students were collected. All teachers taught the focal topic of quadratic equations within the target year groups (year 8 to 11), with the majority (71%) of the students being in school year 10. The mean number of students per class was 23.6 students (SD = 6.50). A total of 58% of the teachers were female and their average age was 35.7 years (SD = 8.40) with an average work experience of 9.9 years (SD = 7.00). Students were 14.8 years old (SD = 0.61) on average, with 54% of them being female. Study participation was voluntary for both teachers and students.

### Measures

#### Teacher Self-Efficacy

##### Generalized Task-Specific Teacher Self-Efficacy

Teachers were asked to rate their self-efficacy beliefs during teaching with a short version of the task-specific TSES devised by Tschannen-Moran and Woolfolk Hoy (2001). The TALIS Video Study had used a shortened version with six items of this TSES to keep the size of the questionnaire manageable (for the items used, refer to the Appendix). The questionnaire included questions about teachers’ self-efficacy beliefs about key teaching tasks such as classroom management, instructional strategies, and student engagement. The introductory stem was “In your teaching in general, to what extent can you do the following?” and the six items were recorded on a Likert-type scale ranging from 1 (*not at all*) to 4 (*a lot*). A sample item was “Help my students value learning.” Cronbach’s alpha for this scale was 0.79.

##### Class-/Task-Specific Teacher Self-Efficacy

A modified version of the task-specific TSES questionnaire that included a class-specific component was also used. The introductory sentence and all items in it referred to a specific class: the introductory stem of the class-/task-specific version was “In your teaching, to what extent can you do the following in the target class?” and the six items were recorded on a Likert-type scale ranging from 1 (*not at all*) to 4 (*a lot*). A sample item was “Help these students value learning.” Cronbach’s alpha for this scale was 0.69.

#### Teaching Quality

The students in each class rated teaching quality in mathematics based on classroom management, cognitive activation, and student support (for the items used, refer to the Appendix). The ratings included items that were adapted from PISA (2003, 2012). All items were rated on a 4-point Likert scale ranging from 1 (*strongly disagree*) to 4 (*strongly agree*). Multilevel McDonald’s omega indices reflect the level-specific reliability of the teaching quality scales (Geldhof et al., 2014).

*Classroom management* was assessed with a 10-item scale, including items about routines, monitoring, and disruptions, e.g., “When the lesson begins, our mathematics teacher has to wait quite a long time for us to quieten down.” Within-level ω was 0.76 and between-level ω was 0.99.

*Cognitive activation* was assessed with a 7-item scale including items about students’ cognitive engagement and participation in discourse, e.g., “Our mathematics teacher presents tasks for which there is no obvious solution.” Within-level ω was 0.71 and between-level ω was 0.87.

*Student support* was assessed with an 8-item scale including items about the student–teacher relationship and teacher support, e.g., “My mathematics teacher makes me feel she/he really cares about me.” Within-level ω was 0.89 and between-level ω was 0.99.

### Statistical Analyses

#### Multilevel Path Analyses

Mplus 8.6 was used for all analyses (Muthén and Muthén, 1998–2017), applying maximum likelihood estimation with robust standard errors (MLR). Missing data were handled with full-information maximum likelihood estimation (FIML), as missing data on all variables were below 5%.

A multilevel path analysis was conducted to account for the hierarchical structure of the data (students nested within classrooms). The three dimensions of teaching quality were included as dependent variables. Measures were based on student ratings, which were combined to manifest scale values per student and subsequently decomposed into within-class level (level 1) and between-class level (level 2) variance components (for advantages of latent aggregation, refer to the study by Lüdtke et al., 2008). For the first research question, manifest z-standardized scales of both TSE scales were used on level 2 as predictor variables. Due to sample size constraints on level 2, we refrained from using latent modeling of TSE and a doubly latent operationalization of teaching quality dimensions and instead used sum scores for the variables.

For the second research question, we used the MODEL CONSTRAINT option to create additional difference parameters to compare the structural paths between the two different TSE scales and teaching quality dimensions. To test the difference parameters against zero, the variances of both predictors on level 2 had to be equal. In order to express the relations in the form of standardized regression coefficients, both the predictors and the criteria were standardized. As Mplus does not standardize the variables separately on both levels when using the DEFINE STANDARDIZE function, both predictors and all three dependent variables were standardized on level 2 by means of a linear transformation within Mplus (subtraction of the level 2 mean, division by the square root of the level 2 variance).

As our hypotheses are directional, one-tailed tests were used with a significance level of *p* < 0.05 (Ruxton and Neuhäuser, 2010; Cho and Abe, 2013). The final model was fully saturated; model fit was therefore trivially perfect.

## Results

### Descriptive Statistics and Correlations

Table 1 reports the means, standard deviations, and intercorrelations for the variables on level 2 (latent mean aggregation of student-rated teaching quality) along with the intraclass correlations (ICC1) and the reliability of the class-aggregated scores (ICC2) for the three teaching quality dimensions.

**TABLE 1:** Descriptive statistics and intercorrelations of the study variables on level 2.

	(1)	(2)	(3)	(4)	(5)
(1) Generalized task-specific TSE	3.44 (0.42)				
(2) Class-/task-specific TSE	0.50**	3.41 (0.39)			
(3) Classroom management ICC(1) = 0.36, ICC(2) = 0.93	0.12	0.26*	2.95 (0.28)		
(4) Cognitive activation ICC(1) = 0.16, ICC(2) = 0.82	0.12	0.10	0.62**	2.81 (0.22)	
(5) Student support ICC(1) = 0.24, ICC(2) = 0.88	0.08	0.21*	0.58**	0.63**	3.15 (0.26)

Mean values and standard deviations of the variables are presented on the diagonal. *p < 0.05; **p < 0.01 (two-tailed).

Results showed that the two TSE scales were highly correlated. The three teaching quality dimensions were also highly intercorrelated. ICC(1) values for the student-rated teaching quality dimensions ranged from 0.16 to 0.36, indicating that between 16 and 36% of the total variance occurred due to systematic between-class differences, supporting the decision to use multilevel analysis. ICC(2) values, which show the degree of consistency in students’ ratings within a class, indicated a high consistency across all three teaching quality dimensions (Table 1).

Class-/task-specific TSE was positively associated with classroom management and student support, whereas generalized task-specific TSE was unrelated to all three teaching quality dimensions.

### Multilevel Path Analyses

**FIGURE 1:**
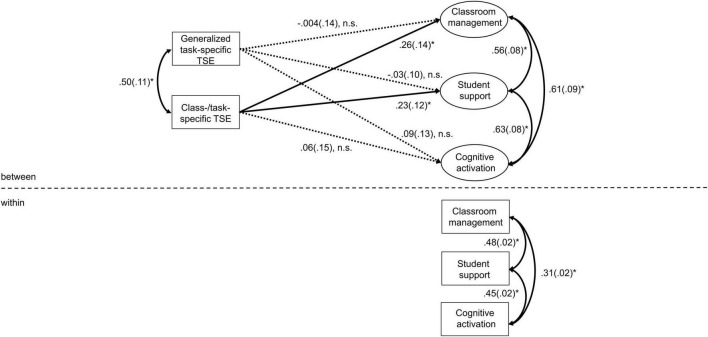
Multilevel path model predicting teaching quality by generalized task-specific and class-/task-specific TSE. Standardized regression coefficients. Saturated model. *p < 0.05 (one-tailed).

The cross-sectional structural paths between class-/task-specific TSE and teaching quality dimensions were tested in a multilevel path analysis. In line with Hypotheses 1a and 1c, class-/task-specific TSE was significantly positively related with classroom management and student support (Table 2 and Figure 1). The relation between class-/task-specific TSE and cognitive activation, however, was not significant. Thus, Hypothesis 1b was not confirmed.

**TABLE 2:** Multilevel path analysis to estimate the associations of the two TSE scales and teaching quality dimensions.

	Classroom management		Cognitive activation		Student support	
	β (SE)	*P*-Value	β (SE)	*P*-Value	β (SE)	*P*-Value
Class-/task-specific TSE	0.26* (0.14)	0.04	0.06 (0.15)	0.35	0.23* (0.12)	0.03
Generalized task-specific TSE	−0.004 (0.14)	0.49	0.09 (0.13)	0.25	−0.03 (0.10)	0.37
Difference parameters	0.26 (0.25)	0.14	−0.03 (0.25)	0.46	0.26 (0.19)	0.08

Standardized coefficients for the reported relations were estimated. *p < 0.05 (one-tailed).

As a next step, we compared the cross-sectional structural paths between class-/task-specific TSE and teaching quality dimensions to the ones with generalized task-specific TSE. From a descriptive perspective, greater positive relations were found between class-/task-specific TSE and classroom management and student support compared with generalized task-specific TSE. This descriptive pattern was not found for cognitive activation, as both TSE scales were unrelated to cognitive activation. Despite this, none of the three pairs of structural paths between both sets of TSE measurements and the teaching quality dimensions differed significantly, as indicated by their corresponding difference parameters (Table 2). Thus, Hypotheses 2a–c were not confirmed.

## Discussion

As previous findings do not provide a clear indication of whether TSE is associated with teaching quality, we aimed to investigate whether a class-specific perspective on TSE, rather than a generalized one, might yield a clearer picture. We followed the often-neglected assumption of social cognitive theory that suggests that TSE measures are not only task-specific but also situation-specific (e.g., class-specific) and most predictive when they are aligned with the behavioral outcome (Bandura, 1997, 2006).

### Relations Between Class-/Task-Specific Teacher Self-Efficacy and Teaching Quality

With our first research question, we investigated the relations between class-/task-specific TSE and teaching quality. Our analyses revealed significant positive cross-sectional relations between class-/task-specific TSE and student-rated classroom management and student support. When teachers felt confident in their teaching capabilities, students rated their teaching quality as higher, resulting in better classroom management and student support. This corroborates with the two existing studies on class-specific adaptions of TSE scales (Holzberger et al., 2013; Perera and John, 2020).

Interestingly, no significant relation was found between class-/task-specific TSE and cognitive activation. This finding might be attributed to the fact that cognitive activation represents a complex and high inference characteristic of teaching quality that requires a higher level of idiosyncratic interpretation and is more difficult to observe than classroom management and student support (refer to e.g., Praetorius et al., 2014). This usually results in a lower agreement between student evaluations, as shown by low ICC values (refer to e.g., Kunter et al., 2008; Fauth et al., 2020; Thommen et al., 2021), which is also true in this study (Table 1) and in lower teacher-student agreement (refer to e.g., Wisniewski et al., 2020) than for classroom management and student support. Students seem to find it more difficult to evaluate cognitive activating teaching reliably. This might explain why the associations between TSE and teaching quality are usually greater and more consistent when teachers instead of students assess their teaching (Lauermann and ten Hagen, 2021). For example, Schiefele and Schaffner (2015) found significant positive relations between TSE and teacher-rated cognitive activation, but none with student-rated cognitive activation. Only a few studies have investigated the relation between TSE and teaching quality from different rater perspectives. We recommend that future studies investigate teaching quality from different perspectives, including, for example, external observer ratings as they are deemed promising (Clausen, 2020). Apart from that, the various conceptualizations and operationalizations of cognitive activation used in previous studies could have also contributed to the inconsistent research findings on the relations between TSE and cognitive activation. In our study, cognitive activation was assessed by two core subdimensions discursive teaching and support of higher-order thinking. However, there are various other approaches to measuring cognitive activation (Praetorius and Charalambous, 2018). Developing a shared understanding of these constructs and their measurement in the research community would benefit the aim of cumulative research on teaching (Charalambous et al., 2021).

The absence of significant relations between TSE and cognitive activation might also be attributed to validity issues: the original TSES is assumed to be conceptually close to teaching quality dimensions, as their underlying sub-dimensions refer to crucial teaching-related tasks (Tschannen-Moran and Woolfolk Hoy, 2001). However, a close analysis of the items of the three sub-dimensions of TSES shows that only TSE for classroom management (“To what extent can you do the following: […] control disruptive behavior in this classroom”) and TSE for student engagement (“[…] get students in this class to believe they can do well in school work”) include aspects similar to the basic teaching quality dimensions of classroom management and student support. In contrast, items of the sub-dimension TSE for instructional strategies such as “[…] use a variety of assessment strategies in this class” relate more strongly to the adaptability and flexibility of a teacher than to cognitive activating teaching. This potential threat to validity caused by a content-related misalignment might therefore have contributed to the absence of a significant relation between the two constructs.

Finally, it might be that teachers’ self-efficacy beliefs only have an indirect predictive effect on (student-rated) cognitive activation. As recently discussed in the review by Lauermann and ten Hagen (2021), it might be that the effect of TSE on student-rated cognitive activation is mediated by teachers’ levels of effort and persistence and classroom processes (e.g., mastery-oriented instructional practices). However, available evidence on direct and indirect effects is scarce and needs further investigation.

### Comparison of the Different Levels of Context-Specificity of Teacher Self-Efficacy Scales and Their Relations With Teaching Quality

With our second research question, we aimed to compare two different context-specific levels of TSE scales directly to get further insight into whether a class- and task-specific TSE scale could be useful for examining the relation with dimensions of teaching quality.

Our findings indicate stronger relations between class-/task-specific TSE and teaching quality than that with generalized task-specific TSE. Significant positive relations between class-/task-specific TSE and classroom management and student support were found. In contrast, no significant relations between teaching quality and generalized task-specific TSE were found.

The difference parameters between the two TSE measures were not statistically significant (Table 2). However, a non-significant *p*-value should be interpreted carefully, as it does not indicate whether there is an actual absence of an effect or possibly a Type II error (refer to e.g., Mehler et al., 2019; Edelsbrunner and Thurn, 2020). It may be that the *p*-values > 0.05 stem from the rather small sample size on level 2 and high standard errors with limited power to find statistically significant effects. To verify if the sample was indeed too small to find significant effects, a power analysis would be appropriate. However, as *post hoc* power analyses are conducted on the basis of sample-based mean differences and conceptually flawed, several researchers advise against conducting such analyses in retrospect (refer to Zhang et al., 2019). Thus, future studies should consider *a priori* power analyses to get information about sample sizes needed to detect statistically significant effects.

Another explanation for the nonsignificant difference parameters might be that the rather low reliability (α = 0.69) of the class-/task-specific TSE might have influenced our findings to some extent. The low reliability stems from the shortened version of the original TSES with only 6 instead of 12 or 24 items. Future studies should preferably use the original scale to ensure higher reliability.

Taken together, the non-significant difference parameters in this study do not yield conclusive information on the added value of class-/task-specific TSE compared with generalized task-specific TSE when examining the relation with teaching quality. Our preliminary findings should therefore be interpreted carefully. Despite the non-significant difference parameters, this study indicates that it makes a difference whether a teacher is asked about his/her self-efficacy beliefs in general or their TSE with reference to a specific class. Both TSE scales seem to be highly correlated (Figure 1), but there seems still enough within-teacher variance that could be explained by contextual factors such as classroom characteristics. This seems in line with the findings of Raudenbush et al. (1992) and Ross et al. (1996) and suggests that teachers’ self-efficacy beliefs should not be treated as generalizable across different classrooms. Assessing TSE with reference to a specific class seems especially important for research in secondary schools or high schools, where a teacher usually teaches more than one class at a time and generalized TSE measures would not indicate which class is being referred to. As our study is the first to specifically investigate different levels of context-specificity of TSE and their associations with teaching quality, further research is needed. As all teachers in our study were only assessed with respect to teaching one particular class, the possibility of a variance decomposition (ICC values) for TSE is not given. It might be interesting for future studies to investigate whether differences in the self-efficacy of a teacher can be identified between different classes. Moreover, it might be interesting to examine which classroom characteristics (e.g., class size, number of students with special educational needs, achievement level, and achievement-related heterogeneity) best explain the within-teacher variance of TSE (refer to e.g., Raudenbush et al., 1992).

### Limitations

The following limitations should be considered when interpreting our findings.

First, our analyses are based on cross-sectional data, which cannot be used to infer causality.^1^ This study was based on the theoretical assumption, drawn from prior studies, that higher self-efficacy beliefs lead to higher teaching quality (Perera and John, 2020). However, from the point of view of social cognitive theory, the relations between the two constructs are reciprocal. As shown by Holzberger et al. (2013), a well-functioning classroom can be interpreted by a teacher as an indicator of achievement and serve as a source of mastery experience, influencing future self-efficacy beliefs. Future studies should therefore use longitudinal data with multiple measurement points to provide clearer information on causal effects between TSE and teaching quality.

Second, the English sample of the TALIS Video Study is not considered representative of the national population of schools, teachers, or students, as voluntary participation led to selective sampling and the number of schools was rather small (OECD, 2020a). The relatively small teacher sample might have led to an underestimation of the variance of TSE and teaching quality and, therefore, of the relations between them. Future studies should replicate our findings with larger samples, to be able to make general conclusions on the added value of a class-specific TSE assessment.

Third, we have examined TSE based on self-assessments because they are best placed to report on their belief in their abilities. However, when interpreting the rather high mean TSE values in our study, methodological biases such as self-desirability or faking should be considered when using self-reports of teacher motivation (Bardach et al., 2021). Following these authors, using complementary measures such as situational judgment tests for TSE evaluations should be considered in future studies.

Lastly, because the shortened version of the TSES had only two items per sub-dimension, in our analyses, we used the total TSE scores to examine the relations between TSE and the dimensions of teaching quality. However, as discussed in section “Relations Between Class-/Task-Specific Teacher Self-Efficacy and Teaching Quality,” stronger relations are expected when predictor and outcome refer to the same entity. There is some evidence to suggest that assessing the relation between matched sub-dimensions of TSE and teaching quality, for example, between TSE for classroom management and student-perceived classroom management is promising (Lazarides et al., 2020). Future research needs to validate our findings with the original version of the TSES and could examine the relations of matched sub-dimensions of TSE and teaching quality separately.

## Conclusion

By adopting a class-specific perspective on TSE, our study aimed to clarify why research findings on the relations between TSE and teaching quality have been inconsistent. Our results suggest significant positive associations between class-/task-specific TSE and student-rated teaching quality. This study is also the first to directly compare different context-specific levels of TSE and their relations with teaching quality. Our results do not provide conclusive information about the added value of the class-/task-specific TSE compared with the generalized task-specific TSE scale. However, based on the descriptive results, it seems promising to continue assessing TSE from a class-specific perspective and replicate our findings with a larger sample. We believe that more consistent use of context-specific TSE scales, as suggested by Bandura’s social cognitive theory, would also help synthesize future research findings.

## Data Availability Statement

The original data reported in this study are publicly available via https://www.oecd.org/education/school/global-teaching-insights-technical-documents.htm.

## Ethics Statement

The study involving human participants was reviewed and approved by the Research Ethics Committee, University of Oxford. Written informed consent to participate in this study was provided by the participants’ legal guardian.

## Author Contributions

DT and A-KP conceived and designed the study. UG and FL provided advice on the study design and analyses. DT carried out the main data analysis and wrote the first draft of the manuscript. UG verified the analyses. A-KP supervised the project. All authors contributed to the article and approved the submitted version.

## Conflict of Interest

The authors declare that the research was conducted in the absence of any commercial or financial relationships that could be construed as a potential conflict of interest.

## Publisher’s Note

All claims expressed in this article are solely those of the authors and do not necessarily represent those of their affiliated organizations, or those of the publisher, the editors and the reviewers. Any product that may be evaluated in this article, or claim that may be made by its manufacturer, is not guaranteed or endorsed by the publisher.

## Footnotes

1 It should be noted that the TALIS Video Study is based on a longitudinal data structure. However, we decided to use teaching quality ratings from the pre-questionnaire for our analyses, as in England, the time interval between pre- and post-data collection was rather short (only around 2 weeks; for further information refer to Ingram et al., 2020). This resulted in very high stabilities for the teaching quality dimensions, which indicate that there was very little time for changes in teaching quality to happen.

## Appendix: Scale Documentation

### Generalized Task-Specific TSE

In your teaching in general, to what extent can you do the following?

1. Get students to believe they can do well in school work.
2. Help my student’s value learning.
3. Craft good questions for my students.
4. Control disruptive behavior in the classroom.
5. Get students to follow classroom rules.
6. Provide an alternative explanation for examples when students are confused.

### Class-/Task-Specific TSE

In your teaching, to what extent can you do the following in the <target class>?

1. Get students in this <class> to believe they can do well in school work.
2. Help these students’ value learning.
3. Craft good questions for these students.
4. Control disruptive behavior in this classroom.
5. Get students in this <class> to follow classroom rules.
6. Provide an alternative explanation for examples in this <class> when students are confused.

### Classroom Management

To what extent do you disagree or agree with the following statements?

1. When the lesson begins, our mathematics teacher has to wait quite a long time for us to quieten down.
2. We lose quite a lot of time because of students interrupting the lesson.
3. There is much disruptive noise in this classroom.
4. In our teacher’s <class>, we are aware of what is allowed and what is not allowed.
5. In our teacher’s <class>, we know why certain rules are important.
6. Our teacher manages to stop disruptions quickly.
7. Our teacher reacts to disruptions in such a way that the students stop disturbing learning.
8. In our teacher’s <class>, transitions from one phase of the lesson to the other (e.g., from <class> discussions to individual work) take a lot of time.
9. Our teacher is immediately aware of students doing something else.
10. Our teacher is aware of what is happening in the classroom, even if he or she is busy with an individual student.

### Cognitive Activation

And how often does your mathematics teacher do the following things?

1. Our mathematics teacher presents tasks for which there is no obvious solution.
2. Our mathematics teacher presents tasks that require us to apply what we have learned to new contexts.
3. Our mathematics teacher gives tasks that require us to think critically.
4. Our mathematics teacher asks us to decide on our own procedures for solving complex tasks.
5. Our mathematics teacher gives us opportunities to explain our ideas.
6. Our mathematics teacher encourages us to question and critique arguments made by other students.
7. Our mathematics teacher requires us to engage in discussions among ourselves.

### Student Support

To what extent do you disagree or agree with the following statements?

1. Our mathematics teacher gives extra help when we need it.
2. Our mathematics teacher continues teaching until we understand.
3. Our mathematics teacher helps us with our learning.
4. I get along well with my mathematics teacher.
5. My mathematics teacher is interested in my well-being.
6. My mathematics teacher really listens to what I have to say.
7. My mathematics teacher treats me fairly.
8. My mathematics teacher makes me feel she/he really cares about me.
